# Recent trends in breast cancer incidence rates by age and tumor characteristics among U.S. women

**DOI:** 10.1186/bcr1672

**Published:** 2007-05-03

**Authors:** Ahmedin Jemal, Elizabeth Ward, Michael J Thun

**Affiliations:** 1 Epidemiology and Surveillance Research, American Cancer Society, 1599 Clifton Rd. NE, Atlanta, GA 30329, USA

## Abstract

**Introduction:**

A recent abstract presented in a breast cancer symposium attributed the sharp decrease in female breast cancer incidence rates from 2002 to 2003 in the Surveillance, Epidemiology, and End Results (SEER) cancer registries of the United States to the reduced use of hormone replacement therapy since July 2002. However, this hypothesis does not explain the decrease that began in 1999 in the age-standardized incidence rate of invasive breast cancer in the nine oldest SEER cancer registry areas, although the trend through 2003 was not statistically significant. In this paper, we examine temporal trends in invasive and *in situ *female breast cancer by age, stage, tumor size, and estrogen receptor/progestin receptor (ER/PR) status in the nine oldest SEER cancer registry areas and consider the implication of these trends in relation to risk factors and screening.

**Methods:**

We performed a joinpoint regression analysis to fit a series of joined straight lines to the trends in age-adjusted rates and described the resultant trends (slope) by annual percentage change (two-sided, *P *< 0.05).

**Results:**

A plot of the age-specific rates of invasive breast cancer shows a decrease in all 5-year age groups from 45 years and above between 1999 and 2003 and sharp decreases largely confined to ER^+ ^tumors in age groups from 50 to 69 years between 2002 and 2003. In joinpoint analyses by tumor size and stage, incidence rates decreased for small tumors (less than or equal to 2 cm) by 4.1% (95% confidence interval [CI], 0.2% to 7.8%) per year from 2000 through 2003 and for localized disease by 3.1% (95% CI, 1.2% to 5.0%) per year from 1999 through 2003. No decrease in incidence was observed for larger tumors or advanced-stage disease during the corresponding periods. Rates for *in situ *disease were stable from 2000 through 2003 after increasing rapidly since 1981.

**Conclusion:**

Two distinct patterns are observed in breast cancer trends. The downturn in incidence rates in all age groups above 45 years suggests a period effect that is consistent with saturation in screening mammography. The sharp decrease in incidence from 2002 to 2003 that occurred in women 50 to 69 years old who predominantly, but not exclusively, had ER^+ ^tumors may reflect the early benefit of the reduced use of hormone replacement therapy.

## Introduction

A recent abstract presented in a breast cancer symposium suggested that the sharp decrease in female breast cancer incidence rates from 2002 to 2003 in the Surveillance, Epidemiology, and End Results (SEER) cancer registries of the United States may have resulted from the reduced use of hormone replacement therapy (HRT) following a July 2002 publication from the Women's Health Initiative [1]. However, the age-standardized delay-adjusted incidence rate of invasive breast cancer in the nine oldest SEER cancer registry areas began to decrease in 1999, although the trend through 2003 was not statistically significant by joinpoint analysis [2]. The recent downturn follows an 18-year period (1980 to 1998) in which breast cancer incidence rates increased by almost 40%. Most of the increase that occurred during the 1980s reflected increased detection of localized disease and tumors measuring less than 2 cm in diameter and has been attributed to the increased use of mammography [3-6]. These effects of mammography are superimposed upon and preceded by long-term birth cohort patterns due to generational changes in reproductive behavior [7,8].

To characterize the recent decrease in breast cancer incidence in relation to earlier secular trends, we examined temporal trends in incidence rates by tumor size, stage, and estrogen receptor/progestin receptor (ER/PR) status and trends for *in situ *breast cancer among women at least 40 years old from 1975 through 2003 by means of incidence data from the nine oldest SEER cancer registry areas. We also examined the age-specific incidence rates for invasive breast cancer by 5-year age intervals. We restricted our analyses to women at least 40 years old because regular mammography screening does not begin before age 40 and HRT use is common after age 50.

## Materials and methods

We obtained the incidence rates for invasive breast cancer from 1975 through 2003 for women by 5-year age intervals beginning at age 40 from the CanQues (Cancer Query Systems) database of the National Cancer Institute (Bethesda, MD, USA), which provides delay-adjusted rates for the nine oldest SEER cancer registries, which are comprised of five states (Connecticut, Hawaii, Iowa, New Mexico, and Utah) and four metropolitan areas (Atlanta, GA; Detroit, MI; San Francisco-Oakland, CA; and Seattle-Puget Sound, WA) [9]. Delay-adjusted rates account for the expected reporting delays and data corrections that most frequently occur in the most recent 1 to 3 years of incidence data (in this case, 2001 to 2003) [10]. We plotted age-specific rates by 5-year age intervals and by year of diagnosis among women at least 40 years old and characterized the trends descriptively without requiring that the recent trend be statistically significant. We restricted our analyses to women at least 40 years old because regular mammography screening does not begin before age 40 and HRT use is common after age 50.

Information on stage at diagnosis, tumor size, and ER/PR status in the nine oldest SEER cancer registries is available for cases diagnosed since 1975 for stage, since 1988 for tumor size, and since 1990 for ER/PR status [11]. We examined the trend in breast cancer incidence rate from 1988 through 2003 according to tumor size for three categories (less than or equal to 2 cm, 2.1 to 3.0 cm, and more than 3 cm) by means of previously published groupings for assessing the influence of early detection and mammography use [3,5]. We also examined the incidence trend according to stage at diagnosis (local, regional, or distant) from 1975 through 2003 and according to ER or PR status from 1990 through 2003. These analyses used the joinpoint model [12] and were restricted to women age 40 and older. A joinpoint regression model fits a series of joined straight lines on a log scale to the trends in age-adjusted rates (2000 U.S. standard population). The resultant trends of varying time periods were described by annual percentage change (that is, the slope of the line segment) (two-sided, *P *< 0.05). Similarly, we analyzed temporal trends in age-standardized incidence rates for *in situ *breast cancer from 1975 through 2003 in women age 40 and older in the nine SEER cancer registry areas by joinpoint analysis. Delay-adjusted data are not available from SEER by stage, tumor size, or ER/PR status or for *in situ *tumors. Therefore, analyses by stage, tumor size, and ER/PR status and of *in situ *tumors were not adjusted for delayed reporting. Incident breast tumors in the SEER database were classified as invasive or *in situ *according to the third edition of the *International Classification of Diseases for Oncology *[13].

## Results

**Figure 1:**
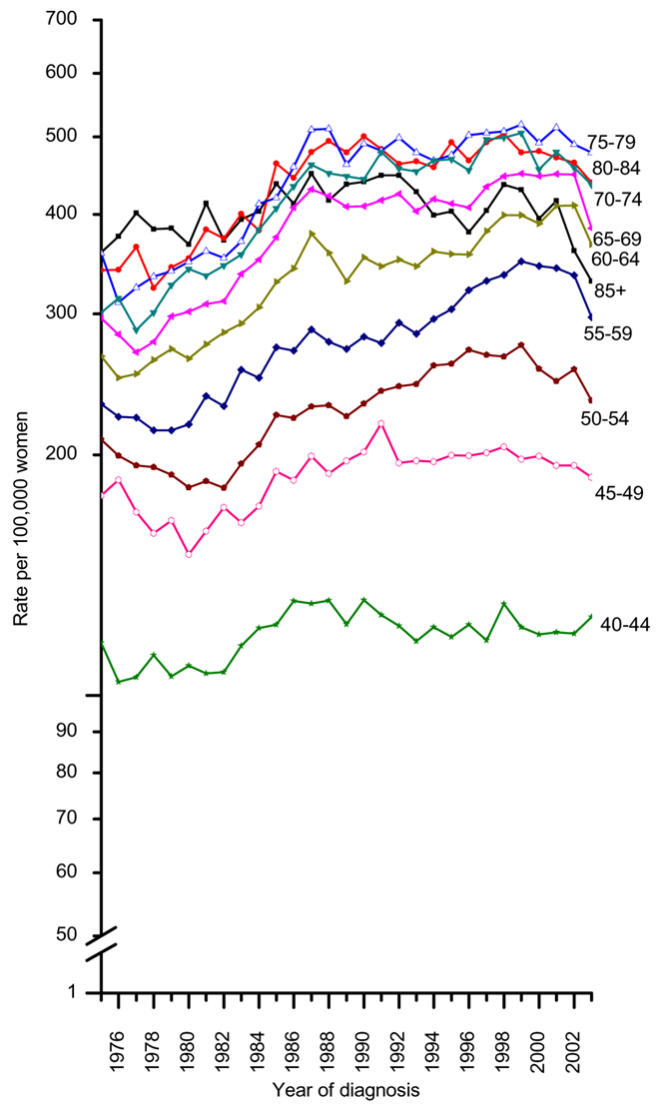
Trends in age-specific invasive breast cancer incidence rates among women 40 years old and above, 1975 to 2003. Rates are adjusted for delay reporting.

From 1975 through 2003, 394,891 invasive and 59,837 *in situ *breast cancer cases were diagnosed in women age 40 and older in the nine oldest SEER cancer registry areas of the U.S. (Table 1). The age-specific incidence rates of invasive breast cancer decreased in every age group of women age 45 and older between 1999 and 2003, although the magnitude and timing of decrease varied by age (Figure 1). Among women less than 60 years old or more than 69 years old, the decrease generally began in 1998 or 1999. In contrast, among women 60 to 64 years old and 65 to 69 years old, all of the decrease occurred from 2002 to 2003, the most recent year for which data are available. The largest percentage decreases from 2002 to 2003 occurred in women 55 to 59 years old (11.3%), 60 to 64 years old (10.6%), and 65 to 69 years old (14.3%).

**Table 1:** Invasive and *in situ *breast cancer cases diagnosed in women 40 years old and above in the nine oldest SEER cancer registry areas of the U.S. (1975 to 2003)

	Invasive		*In situ*	
	
Age (years)	Number	Percentage	Number	Percentage
40–44	28,893	7.3	5,902	9.9
45–49	39,696	10.1	8,719	14.6
50–54	43,107	10.9	8,885	14.8
55–59	45,470	11.5	7,690	12.9
60–64	48,011	12.2	6,878	11.5
65–69	49,652	12.6	7,003	11.7
70–74	47,139	11.9	6,207	10.4
75–79	40,886	10.4	4,689	7.8
80–84	28,378	7.2	2,559	4.3
85+	23,659	6.0	1,305	2.2
Total (≥40)	394,891	100.0	59,837	100.0

SEER, Surveillance, Epidemiology, and End Results.

**Figure 2:**
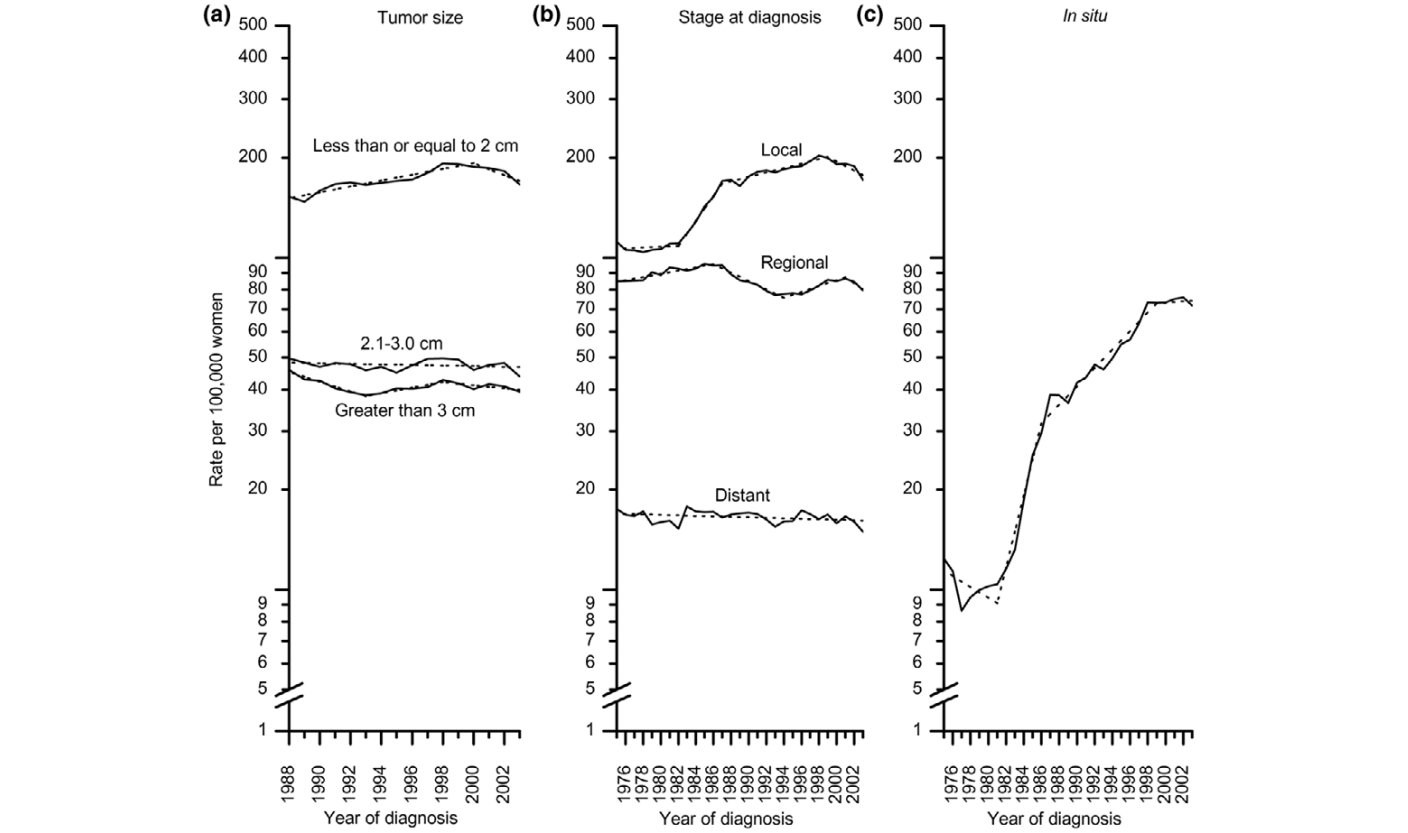
Trends in age-standardized invasive breast cancer rates among women 40 years old and above. **(a) **Trend by tumor size (1988 to 2003). **(b) **Trend by stage (1975 to 2003). **(c) **Trend for *in situ *breast cancer rates (1975 to 2003). Solid lines represent observed rates and dashed lines fitted rates.

In joinpoint analysis by tumor stage and size, the decrease in breast cancer incidence rates was confined to small tumors (less than or equal to 2 cm) and local and regional disease (Figure 2). Statistically significant decreases during the time period 1999/2000 through 2003 were observed for tumors less than or equal to 2 cm (annual percentage decrease, 4.1%; 95% confidence interval [CI], 0.2% to 7.8%) and for localized disease (annual percentage decrease, 3.1%; 95% CI, 1.2% to 5.0%). Incidence rates for *in situ *breast cancer stabilized from 1999 through 2003 after increasing by more than 6.6% (95% CI, 5.6% to 7.6%) per year since 1981 (Figure 2).

**Figure 3:**
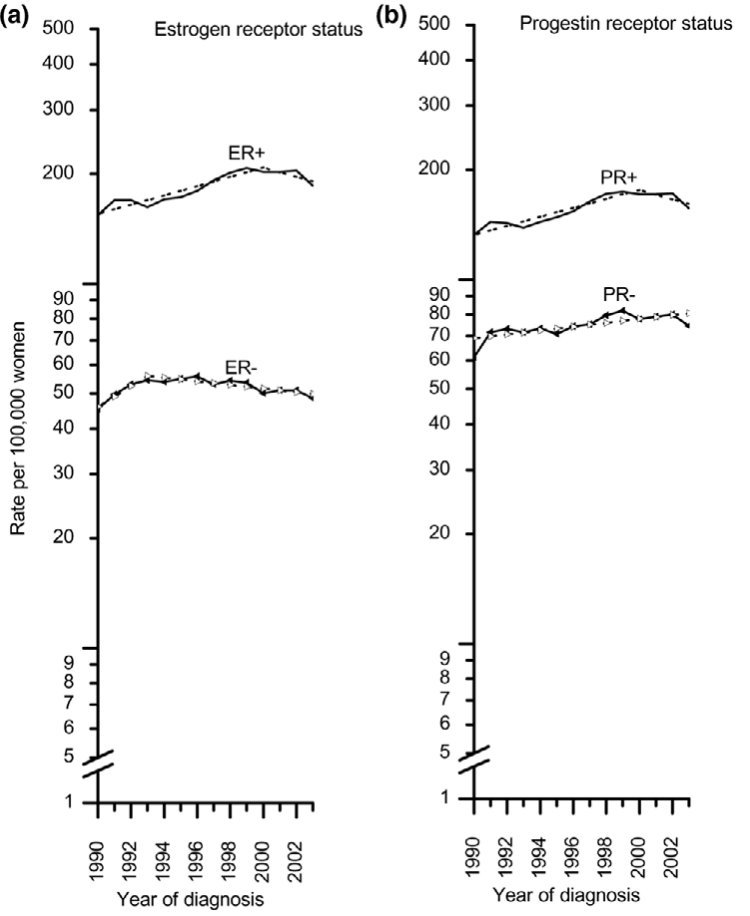
Trends in age-standardized invasive breast cancer incidence rates among women 40 years old and above. **(a) **Trend by estrogen receptor status (1990 to 2003). **(b) **Trend by progestin receptor status (1990 to 2003). Solid lines represent observed rates and dashed lines fitted rates.

Figure 3 shows trends in the age-standardized incidence rates by ER or PR status. Incidence rates for ER^+ ^tumors significantly increased by approximately 3% (95% CI, 2.0% to 3.9%) per year from 1990 to 2000 and then dropped sharply by 9.1% between 2002 and 2003. In contrast, the incidence rates for ER^- ^tumors significantly decreased by 1.1% (95% CI, 0.6% to 1.7%) per year from 1990 to 2003. The decrease was largest (4.8%) between 2002 and 2003. Similar to the incidence rates for ER^+ ^tumors, those for PR^+ ^tumors increased significantly by 2.9% (95% CI, 2.0 to 3.8) per year from 1990 to 2000 and then sharply decreased by 9.1% between 2002 and 2003. The opposite was observed for PR^- ^tumors, which according to joinpoint analysis continued to increase by 1.2% (95% CI, 0.5 to 1.9) per year throughout the entire time interval from 1990 to 2003. However, in the 1-year interval from 2002 to 2003, the incidence rate for PR^- ^tumors decreased by 6.9%. Further analysis by 5-year age interval showed that the decrease in the incidence rates from 2002 to 2003 was much larger in women 50 to 69 years old for ER^+ ^and PR^+ ^than for ER^- ^and PR^- ^tumors. For example, from 2002 to 2003, the incidence rate in women 65 to 69 years old decreased by 20% for ER^+ ^and PR^+ ^tumors compared to an increase of 2% for ER^- ^tumors and a decrease of 9% for PR^- ^tumors.

The SEER data also reflect improvements in tumor staging, measurement, and ER/PR assays over the 30-year duration of the program. The incidence of breast cancer with unknown stage at diagnosis decreased from 13.5 cases per 100,000 in 1975 to 4.9 in 2003 among women age 40 and older. Similarly, between 1988 and 2003, the incidence of tumors of unspecified size decreased from 35.9 cases per 100,000 to 17.5. Between 1990 and 2003, the incidence rate of tumors with unspecified ER or PR status decreased from more than 80 cases per 100,000 in 1990 to approximately 40 cases per 100,000 in 2003.

## Discussion

We find two distinct patterns with respect to recent trends in breast cancer incidence in the nine oldest SEER cancer registry areas. First, a downturn in the incidence rates began in 1998/1999 in all age groups of women 45 years old and above. This pattern is most consistent with a calendar period effect relating to the saturation or plateau in screening mammography as described below. A second trend is the sharp decrease in breast cancer incidence among women 50 to 69 years old between 2002 and 2003 which may represent an early benefit of the reduced use of HRT.

Several aspects of the downturn in incidence rates which began in 1998/1999 are consistent with a period effect due to a plateau in screening mammography. First, the deceases are observed in multiple age groups at roughly the same time and are greatest for small tumors (less than or equal to 2 cm) and localized or *in situ *disease that are most commonly detected by mammography [14]. Second, the timing of the decrease coincides with a plateau in mammography use as measured by national surveys. The percentage of women age 40 and older who report that they had a mammogram within the past 2 years has essentially stabilized since 1999 after increasing from 29% in 1987 [15]. According to data from the National Health Interview Survey [15], this percentage was 70.3% in 1999, 70.4% in 2000, and 69.5% in 2003. Typically, incidence rates decrease when the penetrance of a screening test reaches a plateau due to a reduced pool of undiagnosed prevalent cases.

The rapidity of the decrease in breast cancer incidence rates after the dramatic reduction in the use of HRT which followed the publication of the Women's Health Initiative in July 2002 [16-18] is not inconsistent with the relationship being caused by withdrawal of HRT. Sudden withdrawal of a promoting agent, such as HRT, could slow the growth of tumors that are below the detection limit of mammography and decrease incidence at least in the short term. The observed decrease was largest in women 50 to 69 years old, in whom HRT use is most common, and predominantly (but not entirely) involved ER^+ ^tumors [1]. However, the recent decrease in HRT use cannot account for the reduction in breast cancer incidence that occurred before 2002 or for the decreased incidence in women age 75 and older. If the decrease in ER^+ ^tumors from 2002 to 2003 in women 50 to 74 years old were entirely due to the reduction in HRT use, the maximum HRT contribution to the decrease would be 75%.

Delay-adjusted data are not available by stage, tumor size, or ER/PR status or for *in situ *breast tumors. Therefore, our analyses by stage, tumor size, and ER/PR status and for *in situ *breast tumors did not account for delayed reporting. Delayed reporting would increase rather than decrease the incidence of localized, small, or *in situ *tumors. We reanalyzed the data by stage and tumor size after accounting for delayed reporting, assuming that delayed reporting for each tumor size and stage category was similar to that of all invasive cases combined. In this sensitivity analysis, the decreases in incidence rates over the most recent 4 to 5 data years were only slightly attenuated for tumors less than or equal to 2 cm (from 4.1% to 3.6% per year) and for localized disease (from 3.1% to 2.8%), differences that are too small to invalidate the findings. Delayed reporting would cause our analysis to underestimate the incidence of *in situ *tumors for the most recent 1 to 2 years but cannot account for the incidence plateau that began in 1998. It is also noteworthy that over the study period the improvements in tumor staging, measurements, and ER/PR assays may have contributed in part to the temporal increase in the rates for specified types of breast cancer.

## Conclusion

We observe two distinct patterns in recent trends in breast cancer incidence rates in the nine oldest SEER cancer registry areas. First, the downturn in incidence rates in multiple age groups of women 45 years old and above suggests a period effect that is consistent with saturation in screening mammography. Second, the sharp decrease in incidence from 2002 to 2003 in women 50 to 69 years old, which is larger in ER^+^/PR^+ ^tumors than in ER^-^/PR^- ^tumors, may reflect early consequences of the reduced use of HRT.

## Abbreviations

CI = confidence interval; ER = estrogen receptor; HRT = hormone replacement therapy; PR = progestin receptor; SEER = Surveillance, Epidemiology, and End Results.

## Competing interests

The authors declare that they have no competing interests.

## Authors' contributions

AJ drafted the manuscript and performed the statistical analysis. EW and MJT helped draft and revise the manuscript. All authors read and approved the final manuscript.
